# An unconventional hydrogen effect that suppresses thermal formation of the hcp phase in fcc steels

**DOI:** 10.1038/s41598-018-34542-0

**Published:** 2018-10-31

**Authors:** Motomichi Koyama, Kenji Hirata, Yuji Abe, Akihiro Mitsuda, Satoshi Iikubo, Kaneaki Tsuzaki

**Affiliations:** 1 Department of Mechanical Engineering, Kyushu University, Motooka 744, Nishi-ku, Fukuoka 819-0395 Japan; 2 Graduate School of Life Science and Systems Engineering, Kyushu Institute of Technology, Hibikino 2-4, Wakamatsu-ku, Kitakyushu 808-0196 Japan; 3 Department of Physics, Kyushu University, Motooka 744, Nishi-ku, Fukuoka 819-0395 Japan; 4 HYDROGENIOUS, Kyushu University, Motooka 744, Nishi-ku, Fukuoka 819-0395 Japan

## Abstract

Iron and steels are extensively used as structural materials, and have three primary phase structures: Body-centered cubic (bcc), face-centered cubic (fcc), and hexagonal closed-packed (hcp). Controlling phase stabilities, especially by the use of interstitials, is a universal method that provides a diverse variety of functional and mechanical properties in steels. In this context, hydrogen, which can act as an interstitial species in steels, has been recognized to promote phase transformation from fcc to hcp. However, we here report a dramatic effect of interstitial hydrogen that suppresses this hcp phase transformation. More specifically, the fraction of hcp phase that forms during cooling decreases with increasing diffusible hydrogen content. This new finding opens new venues for thermodynamics-based microstructure design and for development of robust, strong, and ductile steels in hydrogen-related infrastructures.

## Introduction

Hydrogen is a key resource for next-generation green energies^1^, which creates new demands of hydrogen-compatible infrastructures. A challenge to develop such hydrogen-related infrastructures is the significant effect of hydrogen on mechanical degradation of materials^2^. Specifically, the effect of hydrogen in structural steel components causes deterioration of ductility, delayed fracture, and acceleration of fatigue crack growth, all of which are critical problems requiring solutions to realize a hydrogen-energy based-society. Hydrogen-induced mechanical degradation stems from multiple factors such as hydrogen diffusivity/segregation^3^, cohesive energy^4^, number of vacancies^5^, dislocation mobility^6^, crack tip deformability^7^, and phase stability of fcc structures^8–10^. In particular, phase stability has been recognized as a critical factor, because it causes a diffusionless transformation at ambient temperature to bcc or hcp structures. This in turn causes significant changes to hydrogen-related factors such as hydrogen diffusivity^11^. Moreover, solute hydrogen significantly affects the phase stability of fcc^12^, and thus, the synergetic effect of hydrogen and phase stability dramatically alters susceptibility to hydrogen embrittlement^13,14^.

From a viewpoint of phase stability, the diffusionless transformation from fcc to hcp is key to understanding the hydrogen-related phase transformation. More specifically, the hcp phase acts as metastable state of bcc, or in other words, the relative phase stability of fcc compared to hcp affects not only hcp transformation but also bcc transformation in fcc steels^15,16^. In this context, the effect of solute hydrogen on the phase stability of the fcc to hcp has been experimentally investigated for a half-century, and all such experimental studies have indicated that hydrogen promotes hcp transformation^12,17–21^. A possible reason for this is reported to be a reduction in the stacking fault energy^17,20,21^. However, in this study, we unexpectedly in contrast found that solute hydrogen in fact distinctly *suppresses* the hcp diffusionless transformation. This finding rewrites our basic understanding of the hydrogen effect on phase stability of fcc steels, and renovates alloy design strategy for hydrogen-resistant steels. Here we present X-ray diffraction (XRD)-based evidence on this phenomenon, along with a reliable quantification of hydrogen contents.

For this study, we selected a solution-treated Fe-15Mn-10Cr-8Ni (mass%) alloy^22^ for the following four reasons: (1) The initial constituent phase should be fully fcc at room temperature (RT). (2) The estimated Néel temperature should be near or lower than the starting temperature for the hcp diffusionless transformation^23,24^ to avoid magnetic transition effects which can counteract the hcp transformation^24^. (3) The hcp → bcc transformation should not occur after the fcc → hcp transformation. (4) Mobile interstitial elements should not be included to simplify the motion of interstitial hydrogen atoms at ambient temperature. The Fe-15Mn-10Cr-8Ni alloy satisfies these requirements; in particular, the starting temperatures for the hcp diffusionless transformation (M_s_) and its reverse (A_s_) are 244 and 336 K, respectively^25^.

## Results

### Suppression of hcp transformation by hydrogen uptake

**Figure 1:**
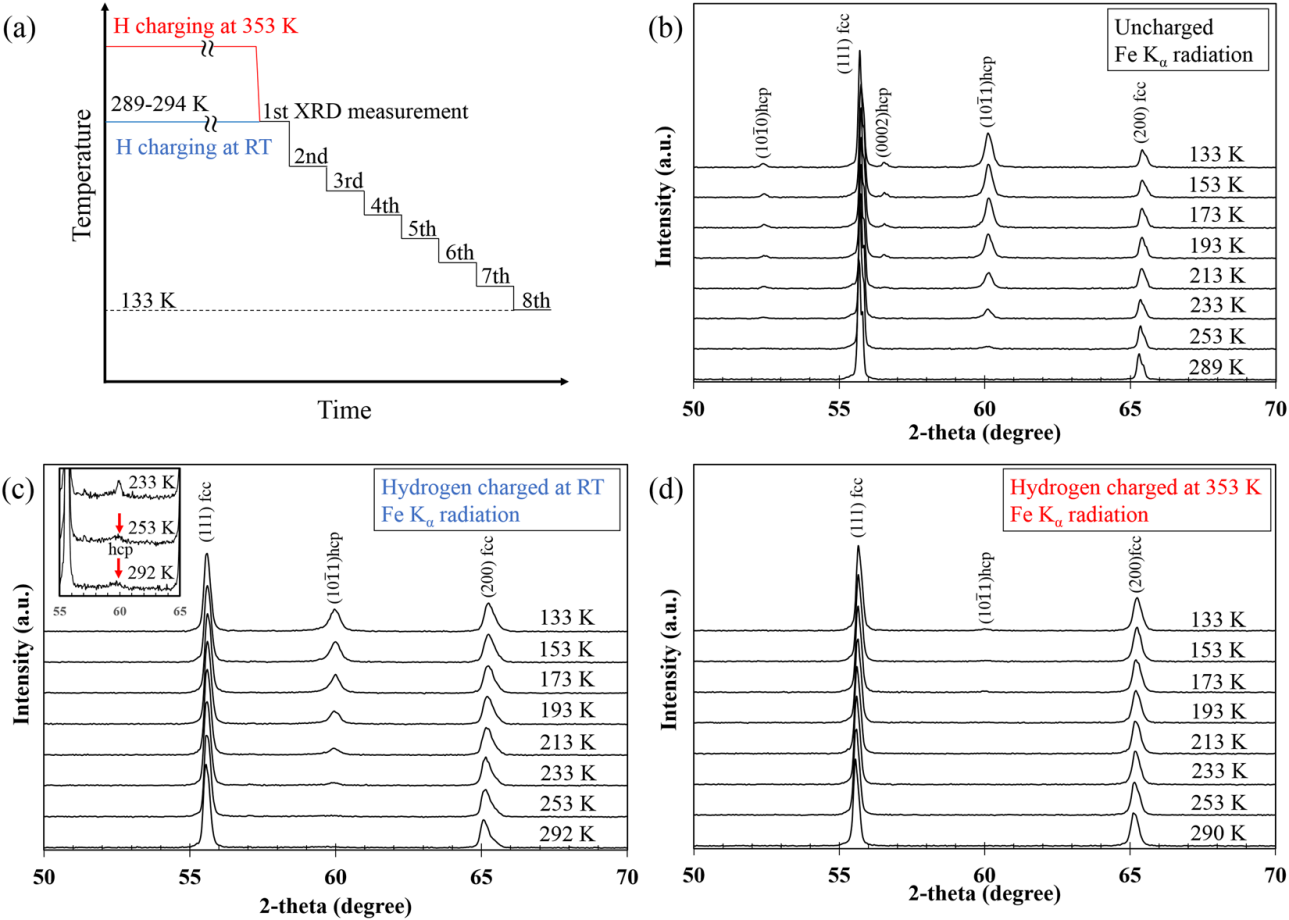
(**a**) Cooling pattern and XRD profiles at each temperature without hydrogen charging (**b**) with hydrogen charging at (**c**) RT and (**d**) 353 K. The inset in (**c**) indicates magnified profiles in the range from 55 to 65 degrees at 233, 253, and 292 K.

**Figure 2:**
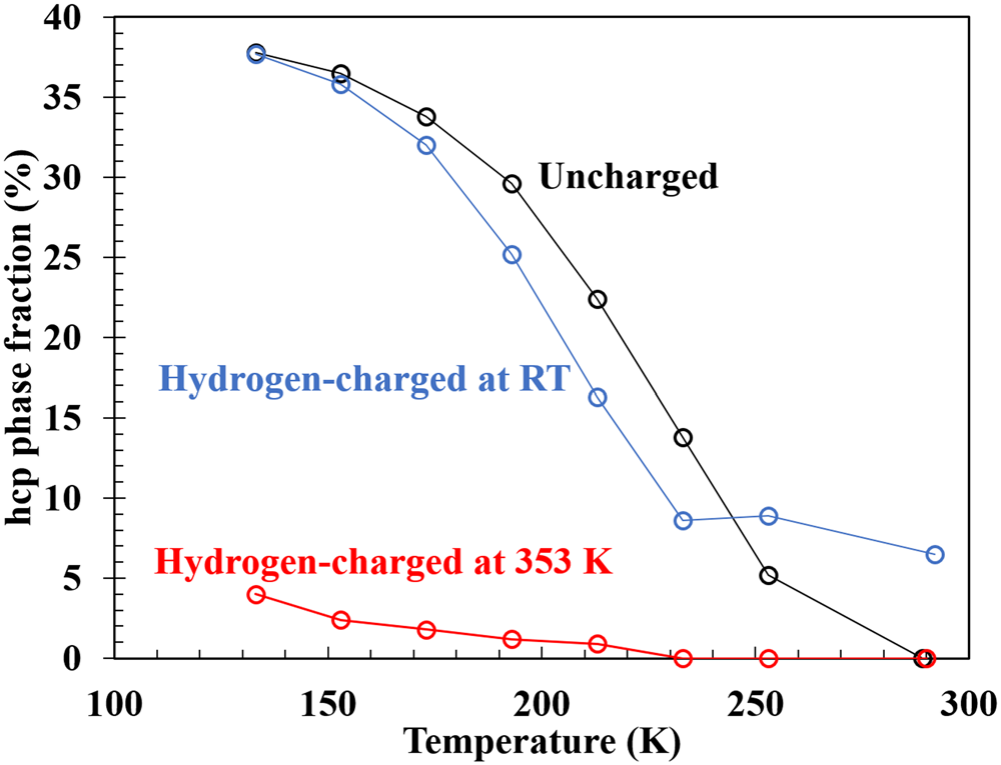
Temperature dependence of hcp phase fraction for different H-charging conditions.

Firstly, we present results of *in situ* cryogenic XRD measurements, following the cooling pattern shown schematically in Fig. 1(a). As shown in Fig. 1(b), the alloy shows a thermally-induced hcp transformation, with the hcp content increasing with decreasing temperature. It is noteworthy that the hydrogen uptake at RT tends to somewhat prevent formation of the hcp phase, comparing the uncharged sample with that in Fig. 1(c). Furthermore, as shown in Fig. 1(d), the specimen with the higher hydrogen content more distinctly exhibits a clear suppression of the thermally induced hcp transformation. Figure 2 summarizes the temperature dependence of the hcp phase fraction present for different hydrogen charging conditions. The pre-existing hcp phase in the specimen hydrogen-charged at RT arises from hydrogen-induced stress occurring during hydrogen charging. Since the temperature of 353 K is higher than the equilibrium temperature of the fcc-hcp transition, no transformation occurs during the hydrogen charging at 353 K. Therefore, below 253 K, the hcp phase fractions decrease with increasing diffusible hydrogen content, irrespective of hydrogen charging temperature. For further clarification, Table 1 shows the diffusible hydrogen content and hcp phase fraction after immersion of the specimens with different hydrogen charging conditions in liquid nitrogen (77 K). It also shows that the hcp phase fraction decreases with increasing diffusible hydrogen content.

**Table 1:** hcp Phase fraction at RT after immersion in liquid nitrogen.

	No charge	Electrochemical charging at RT	Hydrogen gas charging at 473 K	Electrochemical charging at 353 K
Diffusible H content (mass ppm)	0.1	72	82	341
hcp phase fraction	43%	32%	25%	9%

The result post hydrogen gas charging has also been provided for a more general understanding of the hydrogen effect.

### Recovery of hcp transformability with hydrogen desorption

**Figure 3:**
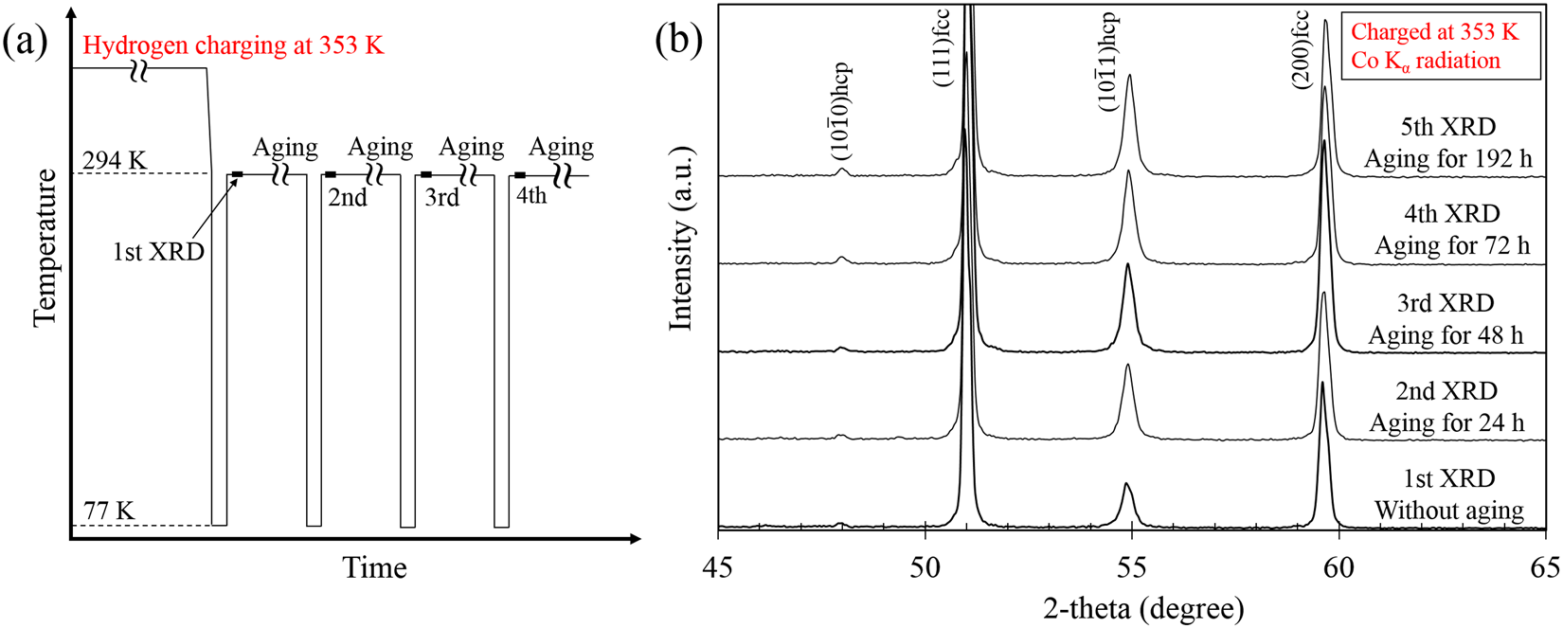
(**a**) Cooling and aging pattern and (**b**) XRD profiles at each cycle with hydrogen pre-charging at 353 K. The aging times given in (**b**) indicate total aging time.

**Figure 4:**
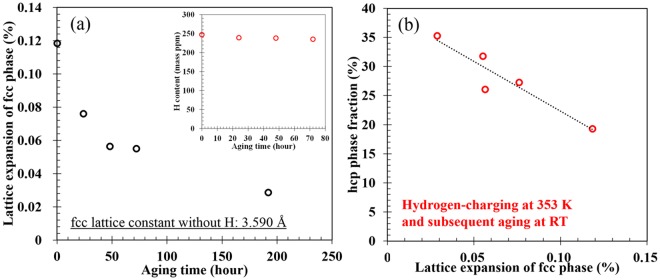
(**a**) Surface lattice expansion of fcc phase varying with aging time. Global diffusible hydrogen contents with different aging times are presented in the inset. (**b**) Relationship between hcp phase fraction and lattice expansion of fcc phase.

**Figure 5:**
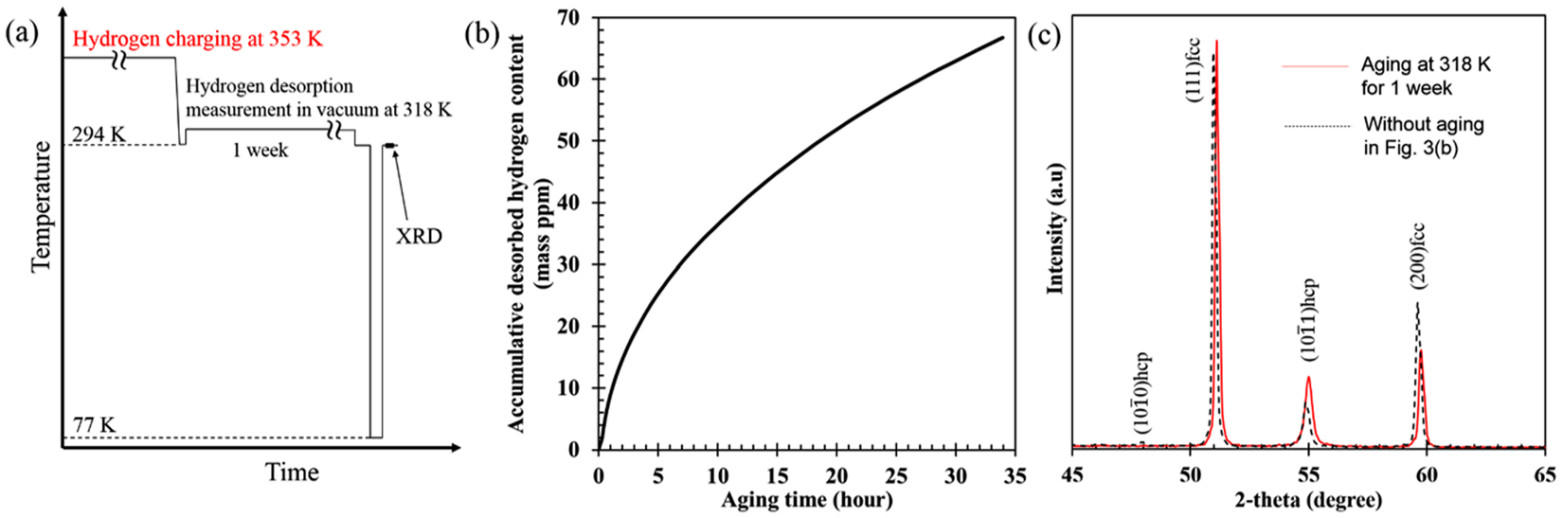
(**a**) Cooling and aging pattern, (**b**) accumulative desorbed hydrogen content, and (**c**) XRD profiles with and without aging at 318 K in vacuum.

Figure 3 shows additional data that support the suppression of hcp transformation. In this set of experiments, the final hydrogen content was controlled by hydrogen desorption via aging at RT, as shown schematically in Fig. 3(a). Here, we performed cyclic aging and cooling treatments to demonstrate the variation in the hcp phase fraction for a single specimen. As expected, the peak intensity corresponding to the hcp phase increases with aging time at RT, Fig. 3(b). Although the global hydrogen content did not significantly change with aging time, the surface lattice expansion attributed to hydrogen decreases as shown in Fig. 4(a). In other words, the decrease in lattice expansion indicates that surface hydrogen is desorbed to a significant extent with aging time. In Fig. 4(b), note that the hcp phase fraction increases with a decrease in the lattice expansion, indicating that the hcp transformation occurs with decreasing surface hydrogen content. Moreover, in order to avoid the cyclic cooling-heating effect, an XRD measurement was also performed after aging for 1 week at 318 K in vacuum without the cyclic process (Fig. 5(a)). Figure 5(b) shows a significant desorption of hydrogen even at the aging time of 34 hours, correspondingly, the XRD profiles shown in Fig. 5(c) indicate recovery of hcp transformability with hydrogen desorption. These facts, taken as a whole, are clear evidence in this study that hydrogen suppresses the hcp transformation in fcc steels.

### Increase in friction stress: hydrogen effects on yield strength and reverse transformation temperature

Diffusionless transformation requires dislocation motion^26^; hence, we must consider a shear stress to drive the dislocations. If hydrogen suppresses hcp transformation via increasing the required shear stress, the effect manifests as an increase in the macroscopic yield strength of the fcc phase. Table 2 shows 0.2% proof stress at 293 K, which generally corresponds to the yield strength. The 0.2% proof stress increased with the hydrogen uptake. Moreover, we note the transformation temperatures with and without hydrogen. The critical transformation temperatures to initiate the forward and reverse diffusionless transformations and their gap are crucial to discuss the hydrogen effect on hcp transformation in terms of thermodynamics and dislocation mechanics. M_s_ decreases by hydrogen charging at 353 K, as indicated in Fig. 2. A_s_ increases by the hydrogen uptake, as shown in Table 2. Thus, hydrogen increases the gap between M_s_ and A_s_.

**Table 2:** Hydrogen effects on yield strength, reverse transformation temperature, and Néel temperature. The Néel temperature is for the fcc phase.

	No charge	Hydrogen gas charging at 473 K	Electrochemical charging at 353 K
Diffusible H content (mass ppm)	0.1	82	341
0.2% proof stress (MPa)	120	138	—
A_s_ temperature (K)	336	349	360
Néel temperature (K)	229	—	223

## Discussion

The *in situ* XRD measurements clearly demonstrate that hydrogen suppresses hcp transformation, which is unexpected behavior compared to that predicted from previous studies. Here we first discuss why these previous studies showed promotion of hcp transformation by hydrogen, raising three points to explain the contradiction. These are in-turn hydrogen-gradient-induced stress, suppression of bcc phase formation, and lastly strain-induced phase nucleation. Regarding the first, hydrogen causes stress during the charging process before the hydrogen distribution attains a homogeneous distribution^27,28^. Therefore, a stress-induced hcp transformation occurs when the hydrogen charging temperature is below the highest temperature for deformation-induced diffusionless transformation^27,28^, as in the case of RT hydrogen charging in the present study. The second point of bcc suppression is related to the fact that bcc transformation occurs via hcp transformation in typical fcc steels such as type 304 stainless steel. Hydrogen has been reported to suppress formation of this bcc phase^29^, and thus, even if hydrogen does not actually promote hcp transformation, the suppression of the bcc phase can apparently increase the hcp phase fraction. Finally, deformation-induced diffusionless transformations can occur by different mechanisms when dislocation re-structuring induces new nuclei^16^. Therefore, a deformation-induced hcp transformation may show different behavior from simple thermally induced hcp transformations. Due to these three reasons, we believe the previous studies could not demonstrate the underlying effect of hydrogen on hcp transformation observed here.

From a viewpoint of mechanism, variations in the friction stress, free energy change, and interfacial energy can affect the behavior of hcp diffusionless transformation. The critical condition for hcp diffusionless transformation has been proposed as follows^26^.

1 $$2n\rho ({\rm{\Delta }}{{\rm{G}}}^{{\rm{fcc}}\to {\rm{hcp}}}+{E}^{str})+2\sigma (n)=-n{\tau }_{0}b$$

where terms on the left and right sides express the thermal driving force and friction stress acting on the transformation dislocation motion at M_s_, respectively. In the equation, *n* is the atomic plane in thickness, *ρ* is the molar surface density along {111}, ΔG^fcc→hcp^ is the free energy change by the transformation from fcc to hcp, *E*^*str*^ is the coherency strain energy, and *σ*(*n*) is the interfacial energy of fcc/hcp. Based on this equation, hcp diffusionless transformation occurs when the thermal driving force is larger than the friction stress. In particular, the free energy change and friction stress are key to understanding the hydrogen effect that suppresses the hcp diffusionless transformation.

**Figure 6:**
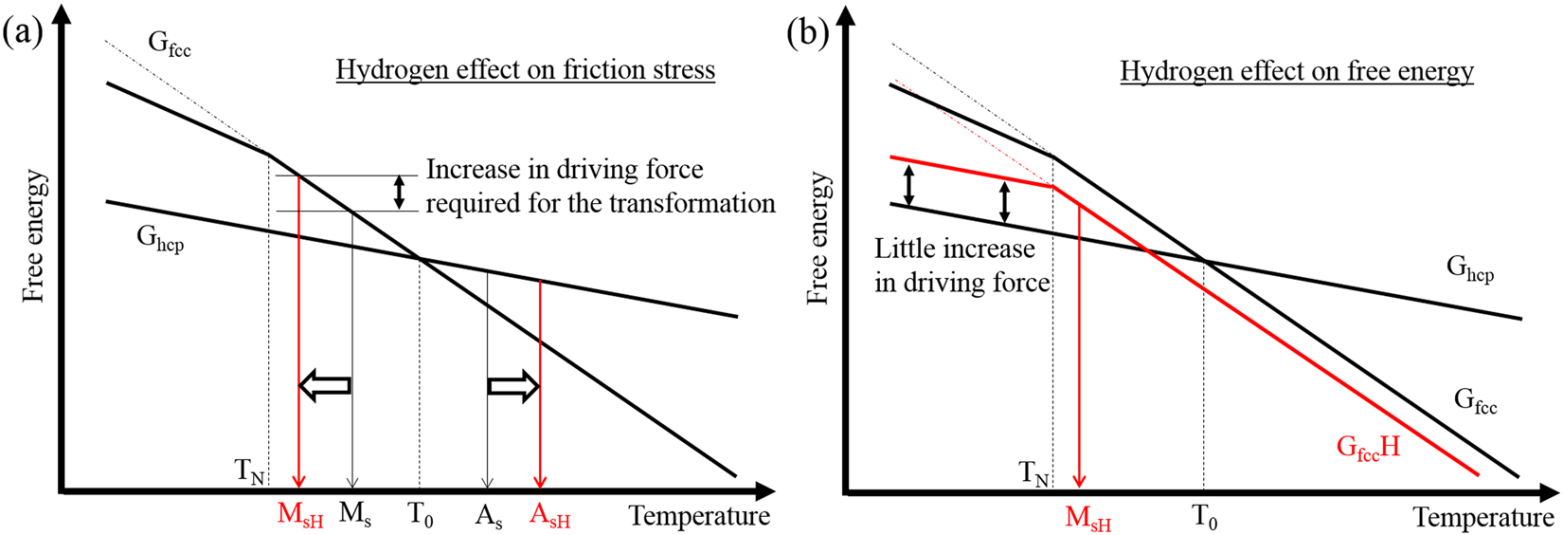
Schematic diagrams of the hydrogen effects on (**a**) friction stress and (**b**) free energy change for the hcp diffusionless transformation. T_0_ and T_N_ indicates an equilibrium temperature of fcc and hcp phases and Néel temperature, respectively. M_sH_ and A_sH_ indicate M_s_ and A_s_ with hydrogen, respectively.

In terms of friction stress, hydrogen increased the yield strength, as indicated in Table 2. Furthermore, hydrogen increased the gap between M_s_ and A_s_, which also implies an increase in the friction stress by hydrogen. These facts indicate that the friction stress for dislocation motion increases by hydrogen. Figure 6(a) schematically shows this hydrogen effect on the friction stress. Hence, a significant factor suppressing the hcp diffusionless transformation is the hydrogen-enhanced friction stress acting on dislocations. However, only the effect of friction stress cannot explain why the transformation rate during cooling of the specimen charged at 353 K is markedly lower than that of the uncharged specimen. In other words, the decrease in hcp transformation rate implies that hydrogen decreases ΔG^fcc→hcp^ below M_s_. In a previous study^30^, an ab-initio calculation indicated that hydrogen can suppress hcp transformation when the hydrogen content reaches 50 at.%. The 50 at.% hydrogen is unrealistic; however, segregation at finite tilt boundaries or stacking fault, which acts as a nucleation site of hcp formation^31^, may locally realize the high concentration of hydrogen. Furthermore, the magnetic transition to antiferromagnatism prevents to increase ΔG^fcc→hcp^ with decreasing temperature^23,24^, and Néel temperature of the present alloy is just below M_s_ as shown in Table 2. The combined effect of the magnetic transition and hydrogen can significantly reduce an increasing rate of ΔG^fcc→hcp^ with decreasing temperature as schematically shown in Fig. 6(b). In summary, the dual effect of hydrogen on the friction stress and free energy suppresses the hcp diffusionless transformation.

## Methods

### Sample preparation

An ingot of the Fe-15Mn-10Cr-8Ni alloy was prepared by vacuum induction melting. The specimen was hot-forged and rolled at 1273 K. The bar was solution-treated at 1273 K and subsequently water-quenched. All specimens were cut by spark machining. All specimens were mechanically polished, followed by electrochemical polishing for final finish.

### Hydrogen charging and measurement of hydrogen content

In this study, diffusible hydrogen was assumed to play a key role on the diffusionless transformation, as it is trapped at interstitial sites, vacancies, dislocations, and grain boundaries that act as nucleation sites of the transformation. The specimens were cathodically charged with hydrogen in a solution of 3%NaCl with a 3 g/l NH_4_SCN at a current density of 50 A/m^2^ at RT for 264 h or 353 K fir 168 h. A platinum wire was used as a counter electrode. The hydrogen charging temperatures were selected to be below and above the A_s_ temperature of 336 K. Accordingly, hcp phase transformation during hydrogen can be avoided at 353 K. In addition, hydrogen gas charging was carried out at 100 MPa at 473 K for 250 h for the following two purposes: (1) for generalizing the present results in terms of hydrogen charging method, and (2) for obtaining a thick specimen with homogeneous hydrogen distribution for tensile testing. The diffusible hydrogen content was measured by thermal desorption spectroscopy (TDS) with a quadrupole mass spectrometer at a heating rate of 400 K/h. Diffusible hydrogen content was defined as cumulative hydrogen content from RT to 573 K. As shown in Fig. S1, saturated hydrogen contents introduced at RT and 353 K with a specimen thickness of 0.2 mm were achieved after 264 and 168 h, respectively. This indicates that the hydrogen distribution in the present hydrogen charging conditions used was homogeneous. Moreover, hydrogen desorption rate at 318 K in the specimen hydrogen-charged at 353 K was measured by holding the temperature for 34 hours in vacuum using the quadrupole mass spectrometer. The temperature of 318 K is the ambient temperature of the chamber. The time of 34 hours corresponds to the limitation of the machine system. The specimen used for the hydrogen desorption experiment was used for an XRD measurement after continued aging at 318 K for 1 week in the TDS chamber.

### XRD measurements

Plate specimens with dimensions of 8 × 8 × 0.2 mm were used for the following XRD measurements. The *in situ* cryogenic XRD measurements were carried out with a Fe target at an acceleration voltage of 40 kV and current of 20 mA. The scanning rate and sampling interval were set to 1 degree per minute and 0.05 degree, respectively. The samples were cooled by a closed-helium-cycle refrigerator, and kept within ± 0.2 K of the desired temperature by an automatic temperature controller during the measurements. The cooling rate was 5 K/min and the temperatures at each step were held for 10 min before starting the measurements. The *ex situ* XRD measurements at RT with the aging process were conducted with a Co target at an acceleration voltage of 40 kV and a current of 20 mA. The scanning rate and sampling interval were set to 1 degree per minute and 0.05 degree, respectively. The specimen for the *ex situ* XRD measurements was cooled by immersing it into liquid nitrogen. The hcp phase fraction was determined by the reference intensity ratio method using integration of (111)_fcc_ and (10-11)_hcp_ diffraction peaks.

### Tensile tests

Tensile tests were carried out at 10^−4^ s^−1^ at RT with and without hydrogen gas charging. The gauge dimensions of the specimen were 4 mm in width, 0.6 mm in thickness, and 30 mm in length. The thickness of 0.6 mm is 10 time larger than the average grain size, which is sufficient for reducing a specimen size effect. However, cathodic hydrogen charging in the aqueous solution requires an unrealistic time to provide homogeneous distribution of hydrogen in the thick specimen. Therefore, hydrogen was introduced to the tensile specimen by exposure to high-pressure hydrogen gas as mentioned above. The strain was determined by a video extensometer.

### DSC measurements

A_s_ temperatures were determined by a differential scanning calorimeter (DSC) operated at a cooling rate of 20 K·min^−1^ using 0.2 mm thick specimens with a square of 2 × 2 mm^2^. The specimen was first cooled from RT to 133 K, and then, heated up to 673 K. A_s_ temperature was defined as the intersection of the tangents of the heat flow peak with the extrapolated base line. Since the transformation rates at M_s_ in the hydrogen-charged specimens were low, the DSC measurement cannot determine M_s_ temperatures. In addition, the transformation temperature of the specimen hydrogen-charged at RT was not measured because of the presence of the stress-induced hcp phase.

### SQUID

In order to measure Néel temperature, magnetic properties were investigated using a superconducting quantum interference device (SQUID) magnetometer in the range of 6–300 K. Thermomagnetization measurements were performed at a cooling rate of 2 K/min under a magnetic field of 5000 Oe.

## Electronic supplementary material

Supplementary materials
