# Supplementary material for: An unconventional hydrogen effect that suppresses thermal formation of the hcp phase in fcc steels
Source: Sci Rep. 2018 Oct 31;8:16136. doi: 10.1038/s41598-018-34542-0 (PMC6208336; doi:10.1038/s41598-018-34542-0)
Supplement: Supplementary file 1 — Supplementary materials [file 41598_2018_34542_MOESM1_ESM.docx]

Supplementary Materials for

An unconventional hydrogen effect that suppresses thermal formation of the hcp phase in fcc steels

Motomichi Koyama*, Kenji Hirata, Yuji Abe, Akihiro Mitsuda, Satoshi Iikubo, Kaneaki Tsuzaki

*correspondence to: Motomichi Koyama at [koyama@mech.kyushu-u.ac.jp](mailto:xxxxx@xxxx.xxx)

Figure S1 shows charging time dependency of diffusible hydrogen content obtained by thermal desorption spectroscopy.


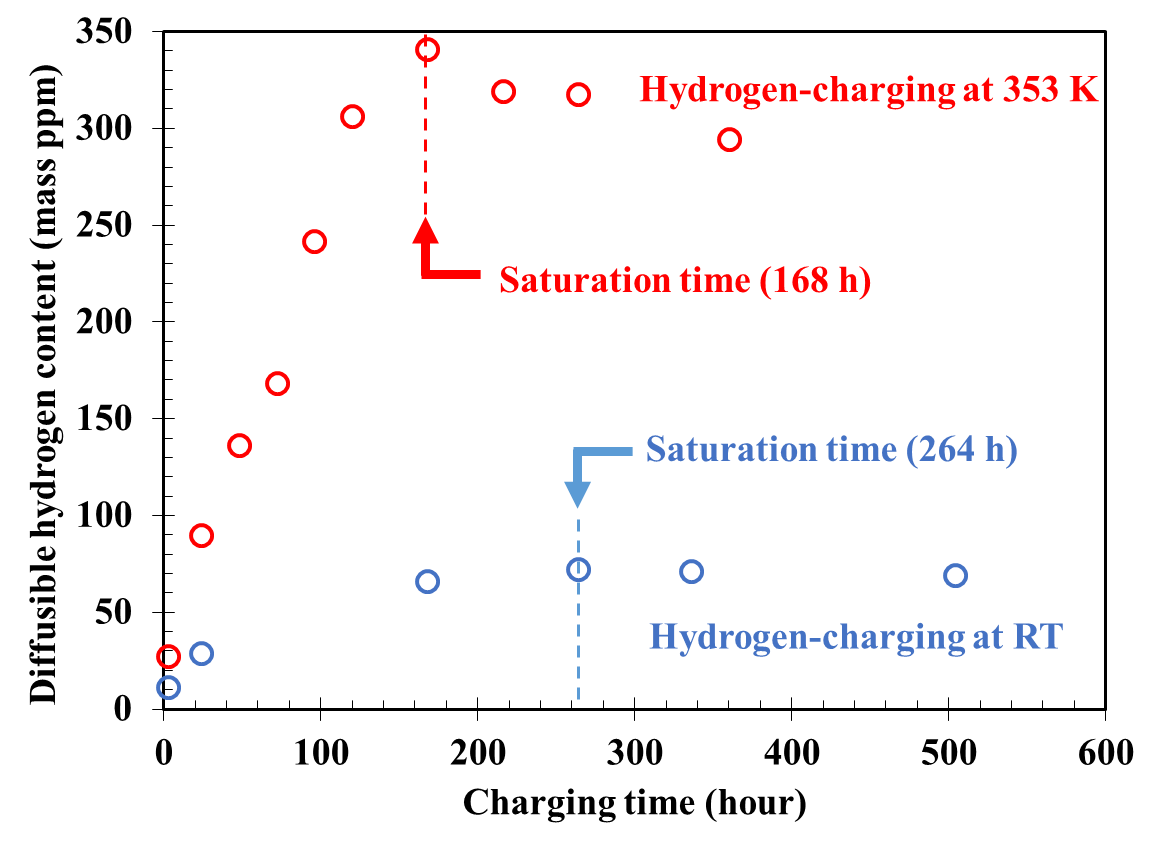


Fig. S1 Diffusible hydrogen content plotted aging changing time.
